# Fast-Scanning Chip-Calorimetry Measurement of Crystallization Kinetics of Poly(Glycolic Acid)

**DOI:** 10.3390/polym13060891

**Published:** 2021-03-14

**Authors:** Yongxuan Chen, Kefeng Xie, Yucheng He, Wenbing Hu

**Affiliations:** Department of Polymer Science and Engineering, State Key Laboratory of Coordination Chemistry, School of Chemistry and Chemical Engineering, Nanjing University, Nanjing 210023, China; dz20240001@smail.nju.edu.cn (Y.C.); DG20240123@smail.nju.edu.cn (K.X.); DG1824029@smail.nju.edu.cn (Y.H.)

**Keywords:** polymer crystallization, crystallization kinetics, thermal analysis, poly(glycolic acid), poly(_L_-lactic acid)

## Abstract

We report fast-scanning chip-calorimetry measurement of isothermal crystallization kinetics of poly(glycolic acid) (PGA) in a broad temperature range. We observed that PGA crystallization could be suppressed by cooling rates beyond −100 K s^−1^ and, after fast cooling, by heating rates beyond 50 K s^−1^. In addition, the parabolic curve of crystallization half-time versus crystallization temperature shows that PGA crystallizes the fastest at 130 °C with the minimum crystallization half-time of 4.28 s. We compared our results to those of poly(_L_-lactic acid) (PLLA) with nearby molecular weights previously reported by Androsch et al. We found that PGA crystallizes generally more quickly than PLLA. In comparison to PLLA, PGA has a much smaller hydrogen side group than the methyl side group in PLLA; therefore, crystal nucleation is favored by the higher molecular mobility of PGA in the low temperature region as well as by the denser molecular packing of PGA in the high temperature region, and the two factors together decide the higher crystallization rates of PGA in the whole temperature range.

## 1. Introduction

Benefitting from its high crystallinity (45–55%), poly(glycolic acid) (PGA) is one of the most extensively applied biodegradable polymers, in particular for bio-medical applications such as sutures and orthopedic parts due to its superior biodegradable, mechanical and gas barrier properties [1,2]. PGA is the simplest aliphatic polyester with a glass transition temperature *T_g_* of about 35–40 °C and a relatively high melting point *T_m_* of about 220–230 °C compared with its analogs, for example poly(lactic acid) (PLA) [3]. The crystalline morphology of PGA was reported by Chatani et al., who determined an orthorhombic space group of $P_{\mathrm{cm n}}$-$D_{2h}^{16}$ system with the lattice dimensions of *a* = 5.22 Å, *b* = 6.19 Å and *c* = 7.02 Å and two molecular chains with the planar zigzag conformation passing through the unit cell forming a sheet structure parallel to the *ac* plane [4]. Montes de Oca et al. proposed that dipolar intermolecular interactions occur between adjacent chains in the crystal unit cell, which may explain the high density (1.69 g/cm^3^) in the crystal of PGA [5]. Moreover, it has been suggested that a weak hydrogen bonding of the C–H···O (ether) exists in PGA microcrystals, which may affect the crystallization behavior and give rise to the unique physical properties of PGA compared to other aliphatic polyesters [6].

Polymer crystallization kinetics mainly determines the crystallinity within a limited time period of industrial processing. It is well-known that the overall crystallization kinetics of polymer is dominated by primary crystal nucleation. According to the classical nucleation theory, the nucleation rate *I* is controlled separately by the activation barrier for short-range diffusion in the low temperature region and the free energy barrier for crystal nucleation in the high temperature region [7], as shown in Equation (1):

(1) $$I=I_{0}exp\left(-\Delta E_{a}/kT_{c}\right)exp\left(-\Delta E_{c}/kT_{c}\right)$$

where ∆*E_a_*/*kT_c_* = *A*/(*T_c_* *– T*_V_) denotes the activation barrier for polymer diffusion in which the Vogel temperature *T*_V_ is about 50 °C below the glass transition temperature *T_g_*; *A* is a constant; *T_c_* is the crystallization temperature; and *k* is the Boltzmann’s constant. $\Delta E_{c}{=8\pi \sigma }^{2}\sigma_{e}{\left[T_{m}^{0}/\Delta H_{c}/\left(T_{m}^{0}{-T}_{c}\right)\right]}^{2}$ represents the free energy barrier for primary crystal nucleation supposed in a cylindrical bundle, with *σ* and *σ*_e_ the surface free energy densities separately on the lateral and folding-end surfaces of lamellar polymer crystals, $T_{m}^{0}$ the equilibrium melting point, and ∆*H*_c_ the heat of fusion for 100% crystalline phase. The activation barrier for short-range diffusion becomes higher at lower temperatures, while the free energy barrier for primary crystal nucleation becomes higher at higher temperatures, leading to a parabolic curvature of overall crystallization rate (or crystallization half-time) versus crystallization temperature with its maximum (or minimum in the case of half-time) in the middle temperature between *T*_g_ and $T_{m}^{0}$. Many studies have considered the isothermal crystallization behaviors of PGA based on techniques such as conventional DSC [8,9] and IR [10]. However, PGA exhibits a rather quick crystallization, thus traditional techniques cannot avoid crystallization during cooling to low temperatures, resulting in the study of isothermal crystallization kinetics of PGA mainly in the high temperature region.

Recently, fast-scanning chip-calorimetry (FSC and its commercial version, Flash DSC1) measurement allows for high heating rates up to 10^5^ K s^−1^, and it has been proven as a powerful tool to characterize the crystallization kinetics down to the low temperature region [11,12,13,14] for a large variety of polymers such as polyethylene [15,16], isotactic polypropylene [17,18] and polyamide [19,20,21]. The non-isothermal and isothermal crystallization behavior of poly(_L_-lactic acid) (PLLA) homopolymer with a mass-average molar mass 120 kDa characterized via Flash DSC was reported by Androsch et al. [22,23], suggesting that −0.5 and −50 K s^−1^ are separately the critical cooling rates to suppress crystallization and nuclei formation upon cooling. Furthermore, the maximum crystallization rate of PLLA is around 110 °C, with the minimum crystallization half-time of 150–200 s. 

Derived from renewable sources, PLA is one of the most commercially successful bioplastics, mainly used in packaging, commodity materials and biomedical sectors owing to its superb processability, biodegradability and optical and mechanical properties [24,25,26]. In fact, PLA represents a complex polymer ascribed to two optically active forms of lactic acids: _L_-lactic acid and _D_-lactic acid. Poly(_L_-lactic acid) (PLLA) is a commercially acceptable product with _L_-lactic acid as the primary yield in the fermentation process. PLLA exhibits a glass transition temperature *T*_g_ of about 55–60 °C [27] and a melting point *T*_m_ of about 175–180 °C [28]. Depending on the crystallization conditions, three different crystalline modifications of PLLA have been formed. PLLA forms the most common α-crystals with 10_3_ helical chain conformation [29,30] upon crystallization above 120 °C, while dominant α’-crystal with a crystal structure similar with α-crystal occurs upon crystallization below 100 °C [31,32,33]. β-crystal and γ-crystal with 3_1_ helical chain conformation can be separately obtained via hot-drawing of melt- or solution-spun fibers [34,35] or via epitaxial crystallization on hexamethylbenzene [36].

**Figure 1:**
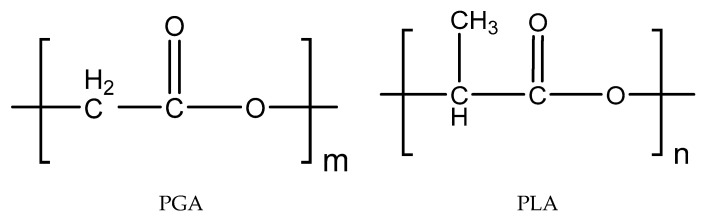
Schematic illustration of the chemical structures of poly(glycolic acid) (PGA) and poly(lactic acid) (PLA).

PGA and PLLA share similar molecular structures, as shown in Figure 1, but they exhibit distinctly different crystallization behaviors. PGA does not have the methyl side group, which is replaced with a much smaller hydrogen side group. This fact reduces its internal rotation barrier of side groups around the backbone chain and thus offers a high molecular mobility of polymer chains. On the one hand, this fact enhances the density of molecular packing in the crystalline phase and thus brings a strong thermodynamic driving force for crystallization. Therefore, compared to PLLA, PGA holds a relatively low glass transition temperature due to its low steric barrier for internal rotation. On the other hand, PGA has a relatively high melting point and large fusion enthalpy due to its compact molecular packing in the crystals. Both factors imply that PGA crystallizes more quickly than PLLA in the entire temperature range from the glass transition temperature to the melting point.

In this work, by means of Flash DSC measurement, we characterized the isothermal crystallization kinetics of PGA in the proper temperature range. We compared our results for PGA to those of PLLA with a similar molar mass to investigate the effect of their molecular structures on the crystallization kinetics. The results confirm that PGA exhibits faster crystallization than PLLA in the entire temperature range.

## 2. Materials and Methods

### 2.1. Materials

PGA granules (molecular weights around 169 kDa, index of polydispersity 16, *T*_m_ ≈ 228 °C) were kindly supplied by Shanghai Pujing Chemical Industry Co. Ltd, China, with the batch number 190119. The equilibrium melting point of PGA was reported as 231.4 °C and the heat of fusion for 100% crystalline of PGA was 183.2 J g^−1^ [37].

### 2.2. Fast-Scanning Chip-Calorimetry Measurement

Flash DSC1 (Mettler-Toledo AG, Switzerland) installed with Huber TC-100 intracooler was employed to perform experiments in the temperature range from −100 to 240 °C with maximum heating and cooling rates of 3000 K s^−1^. The raw materials were first cut into a thin film section with smooth top and bottom surfaces using a scalpel, and then, under the equipped optical microscope, they were cut into 20 μm × 20 μm squares with smooth lateral surfaces. Finally, specimens with a typical mass of about 100 ng were prepared. Note that the apparent heat capacity of PGA sample at 250 K was measured by Flash DSC1 at a typical rate of 1000 K s^−1^ as 1.2 × 10^−7^ J K^−1^, while the specific heat capacity of PGA at 250 K (ca. −23 °C) was reported by Wunderlich et al. as 60 J mol^−1^ K^−1^ [38]; thus, our PGA sample mass was estimated as 116 ng from their ratio [39]. By utilizing a string of hair, the specimen was then transferred to the center area of the chip sensor. Before that, the chip sensor had already been conditioned and corrected in accordance with the standard protocol and casted with thin oil film for a good contact between sample and sensor. Afterward, under a constant nitrogen flow at the rate of 50 mL min^−1^, the positioned specimen was slowly heated to a temperature above its equilibrium melting point, ensuring a good thermal contact between the sample and the chip sensor.

After the above preparation of samples, three crystallization experiments were conducted: (1) cooling experiment; (2) heating experiment; and (3) isothermal experiment. The first cooling experiment aimed at determining the critical cooling rate for prohibiting melt crystallization on cooling. The subsequent heating experiment was intended to obtain the critical heating rate for suppressing the cold crystallization on heating. The isothermal experiment was performed upon fast cooling and heating with rates far beyond the critical conditions determined in the first two steps. Completing these three steps, we obtained the temperature dependence of the crystallization half-times of PGA sample to compare the crystallization kinetics with that of PLLA. Since the equilibrium melting point of PGA is 231.4 °C, we chose a hold at 240 °C with the residence time of 0.2 s to remove the thermal history as well as avoid thermal degradation in the sample. As is usual, the heat flow rates were normalized by the heating or cooling rates into the apparent heat capacity in our presentation of results.

## 3. Results

### 3.1. Cooling Experiment

**Figure 2:**
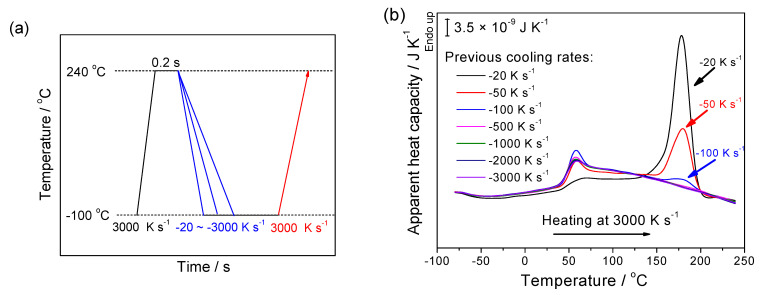
(**a**) Illustration of the temperature program to find the critical cooling rate of PGA to prohibit crystallization process during cooling; and (**b**) apparent heat capacity of PGA as a function of temperature on the heating scans at the rate of 3000 K s^−1^ after cooling at the various rates from −20 to −3000 K s^−1^ depicted in (**a**).

Prior determination of the critical cooling rate is absolutely necessary for isothermal crystallization experiments, which require a rapid cooling process before the target temperature is reached to avoid melt crystallization and even primary crystal nucleation. To obtain the critical cooling rate for suppressing crystallization and primary nucleation on cooling, the sample was first heated to 240 °C and held for 0.2 s to obtain a melt without any thermal history. Afterwards, the sample was cooled to −100 °C at various rates ranging from −20 to −3000 K s^−1^, as illustrated in the temperature program of Figure 2a. Since the sample mass was small, we did not see significant crystallization peaks on the cooling curves. Therefore, heating runs were performed at an extremely high heating rate of 3000 K s^−1^ to avoid cold crystallization on heating, making it possible to measure the crystallinity harvested on cooling based on the integration of melting peaks on the subsequent heating curves. Figure 2b summarizes the heating curves after cooling with various cooling rates. One can see clearly that both glass transition at around 50 °C and melting peaks at around 175 °C occurred. In contrast to the 35–40 °C in conventional DSC, PGA exhibits a rather high glass transition temperature around 50 °C, which is attributed to the specific kinetics of the transition process, namely an increase in *T*_g_ with increasing heating rates. Conversely, the lower melting temperature of around 175 °C, in comparison to 228 °C in conventional DSC, reflects the less stable crystals formed at higher cooling rates since the time available for crystal perfection is greatly reduced. With the increase of cooling rates, the corresponding melting peaks on subsequent heating curves vanishes and the heating curves exhibit an excellent repeatability once the previous cooling rates exceed −100 K s^−1^. Hence, −100 K s^−1^ is the critical cooling rate to prohibit melt crystallization and crystal nucleation on cooling for PGA. The critical cooling rate to suppress melt crystallization of PGA (−100 K s^−1^) is significantly higher than that reported for PLLA (−0.5 K s^−1^) [22], suggesting PGA has a better crystallization capability than PLLA during the cooling process.

### 3.2. Heating Experiment

**Figure 3:**
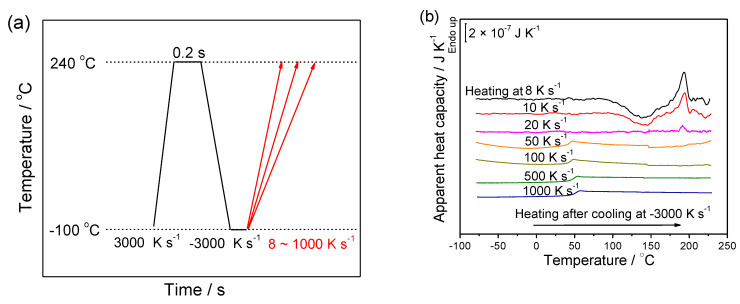
(**a**) Illustration of the temperature program for determining the critical heating rate of PGA to prohibit cold crystallization during the heating process; and (**b**) the apparent heat capacity of PGA as a function of temperature on heating at the various heating rates from 8 to 1000 K s^−1^ after cooling from a hold of 0.2 s at 240 °C at the cooling rate of −3000 K s^−1^ depicted in (**a**).

To determine the critical heating rate for preventing cold crystallization on heating, the heating experiment was performed according to the temperature profile illustrated in Figure 3a. First, the sample was heated to 240 °C and held for 0.2 s to erase the thermal history, followed by a cooling scan at −3000 K s^−1^. Afterwards, the sample was heated to 240 °C at various rates ranging from 8 to 1000 K s^−1^. As shown by the heating curves in Figure 3b, above the glass transition temperature of around 50 °C, the cold crystallization peaks around 140 °C and the melting peaks around 180 °C disappear when the heating rate exceeds 50 K s^−1^, which implies it is the critical heating rate to prohibit cold crystallization of PGA on heating. This critical heating rate (50 K s^−1^) for PGA is much higher than the one reported for PLLA (1 K s^−1^) [33].

### 3.3. Isothermal Experiment

**Figure 4:**
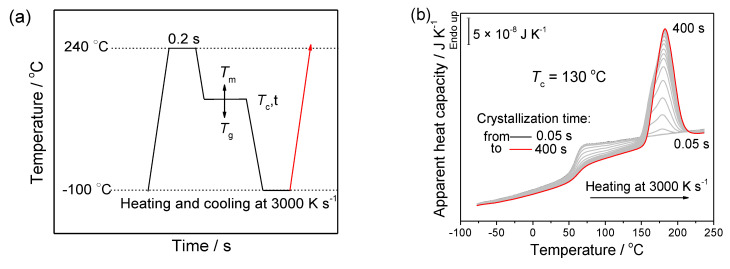
(**a**) Illustration of the temperature program for isothermal crystallization at various crystallization temperatures between *T*_g_ and *T*_m_; and (**b**) the apparent heat capacity of PGA as a function of temperature on heating at the heating rate of 3000 K s^−1^ after isothermal crystallization at 130 °C for the various crystallization times from 0.05 to 400 s depicted in (**a**).

After determining the critical cooling rate to suppress melt crystallization and the critical heating rate to prohibit cold crystallization, we chose high enough cooling and heating rates to carry out an isothermal crystallization experiment. The sample was heated to 240 °C and held for 0.2 s to eliminate the thermal history. Then, the melt was cooled to various crystallization temperatures (80, 90, 100, 110, 120, 130, 140, 150, 160 and 170 °C) that were higher than *T*_g_ but lower than *T*_m_. After staying at the crystallization temperature *T*_c_ for different time periods for isothermal crystallization, the samples were cooled to −100 °C and heated back to 240 °C. It is worth noting that, in this experiment, all the absolute heating and cooling rates were 3000 K s^−1^, well beyond the critical heating and cooling rates of PGA in order to effectively suppress the crystallization process. The detailed temperature protocol of the isothermal experiment is illustrated in Figure 4a.

Weakly double melting endotherms occur when *T*_c_ is lower than 130 °C (see the Supplementary Materials). One can observe that at 100 °C the low-temperature peaks gradually shift to higher temperatures upon the increase of isothermal crystallization periods, implying the formation of more uniform and thicker crystals. Meanwhile, the high-temperature peaks remain constant with the increase of isothermal crystallization periods, suggesting a melt–recrystallization mechanism for the occurrence of double-melting peaks [9]. We took the total melting enthalpy of double-melting peaks into account.

**Figure 5:**
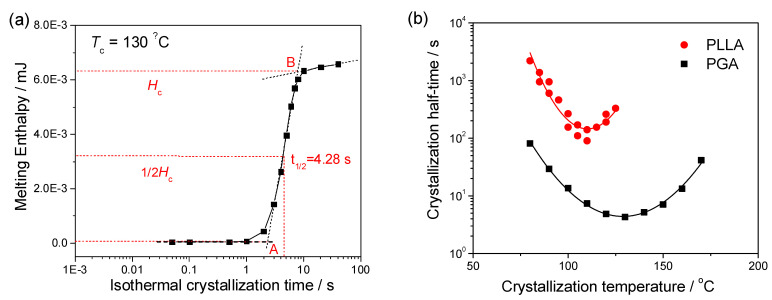
(**a**) The time evolution of crystallization enthalpy on isothermal crystallization of PGA at 130 °C. The crystallization half-time is read as the vertical dashed line. (**b**) The temperature dependence of crystallization half-time of PGA and PLLA [23] during isothermal crystallization process at various temperatures.

We take *T*_c_ = 130 °C as an example to explain the kinetic analysis of PGA isothermal crystallization. According to the cooling and heating experiments, the cooling rate of −3000 K s^−1^ and the heating rate of 3000 K s^−1^ are fast enough for PGA to prohibit the crystallization during the cooling and heating processes. The melting enthalpy ∆*H*_m_ obtained by integrating the melting peak in the heating scan represents the enthalpy change of isothermal crystallization ∆*H*_c_. By evaluating the melting enthalpy from the melting peaks in Figure 4b, we obtain the enthalpy of isothermal crystallization at 130 °C as a function of the crystallization time, as shown in Figure 5a. The curve in Figure 5a exhibits the typical shape of a crystallinity curve for isothermal crystallization. The starting and ending crystallinity during the isothermal crystallization are defined as A and B to obtain the crystallization half-time at 1⁄2 ∆*H*_c_ [19,20], namely 4.28 s for PGA on isothermal crystallization at 130 °C. In the Supplementary Materials, we present all the data treatment of PGA at various crystallization temperatures, e.g. *T*_c_ = 130 °C.

Figure 5b summarizes the crystallization half-times of PGA at various crystallization temperatures from 80 to 170 °C as well as those previously reported for PLLA for the same temperature scales [23]. The parabolic curve of PGA indicates the valid application of the classical nucleation theory on the crystallization kinetics, where the activation barrier for short-distance diffusion and the free energy barrier for crystal nucleation dominate the primary nucleation rate separately in the low- and high-temperature regions. Furthermore, the maximum crystallization rate of PGA occurs at around 130 °C with the minimum crystallization half-time of 4.28 s. In contrast, PLLA crystallizes the fastest around 110 °C and the corresponding crystallization half-time is within 150–200 s. In comparison, the crystallization half-times of PGA are between 4 and 90 s, while those of PLLA range 90–3000 s. Hence, the crystallization of PGA appears distinctly faster than that of PLLA at the same temperatures in the entire temperature range, which confirms our theoretical expectation according to their chain-unit difference.

## 4. Discussion

According to the classical nucleation theory of PGA, the crystallization rate is predominantly controlled by supercooling away from the equilibrium melting point in the high temperature region. The $T_{m}^{0}$ of PGA is reported as 231.4 °C [37], which is higher than that of PLLA (207.0 °C) [40,41] due to the tight chain packing of PGA crystals. Therefore, PGA crystallizes more quickly than PLLA in the high temperature region owing to its higher supercooling from the equilibrium melting point. Conversely, the crystallization rate is primarily dominated by the temperature deviation from the glass transition temperature in the low-temperature region. As discussed above, PGA has a relatively lower glass transition temperature than PLLA, due to the lower steric hinderance upon the internal rotation of the side groups around the backbone chain of PGA. Thus, PGA crystallizes more quickly than PLLA in the low-temperature region as a result of a higher crystallization temperature deviation from the glass transition temperature.

**Figure 6:**
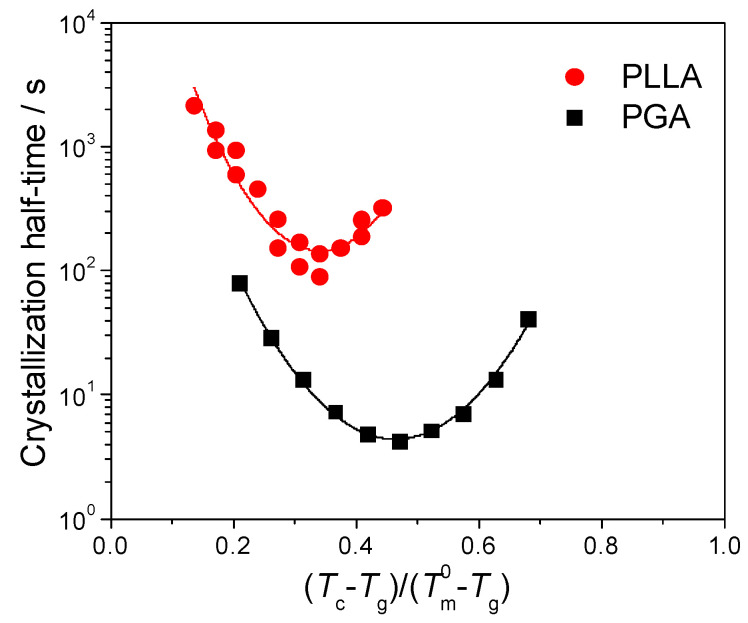
Temperature dependence of crystallization half-time of PGA and PLLA in the relative temperature scales between the glass transition temperatures and the equilibrium melting points, where the *T*_g_ of PGA and PLLA were chosen as 40 and 60 °C, respectively, while the $T_{m}^{0}$ were 231.4 [37] and 207.0 °C [40,41], respectively.

To further investigate the effect of molecular structures on the crystallization kinetics, the crystallization temperatures in Figure 5b were rescaled according to the relative values between the glass transition temperatures and the melting points of PGA and PLLA, respectively. The rescaled crystallization kinetic curves are shown in Figure 6. One can see more convergence between PGA and PLLA curves at the lower end of the temperature range after rescaling, but it is not significant at the higher end.

At high temperatures, the crystallization kinetics is dominated by the relatively high free energy barrier for crystal nucleation, which is connected with the surface free energy *σ* and *σ*_e_, equilibrium melting point $T_{m}^{0}$ and the heat of fusion ∆*H*_c_ according to the rationale of Equation (1). It is reported that the ∆*H*_c_ of PGA and PLLA are 183.2 [37] and 143 J g^−1^ [42], respectively. Therefore, the heat of fusion of PGA is larger than that of PLLA, which contributes to a lower free energy barrier for crystal nucleation of PGA as well. In the low-temperature region, the primary nucleation rate *I* is dominated by the relatively high activation barrier for short-range diffusion, which is correlated with glass transition temperature *T*_g_ and the diffusion constant *A* according to Equation (1). In particular, the temperature dependence of PGA appears relatively stronger than that of PLLA at low temperatures, implying a higher diffusion constant A for PGA.

Overall, in the whole temperature range, PGA apparently crystallizes more quickly than PLLA. This comparison between PGA and PLLA is a good example illustrating the effect of molecular structures on the crystallization kinetics over an entire temperature range.

## 5. Conclusions

Fast-scanning chip-calorimetry has the advantage of tracing the fast crystallization of PGA, in particular at low temperatures. Although fast scanning of temperatures brings thermal lag in the sample, which slightly shifts the crystallization/melting temperatures, it has little influence on their enthalpies for our judgment of critical cooling/heating rates and crystallization half-time. We performed fast-scanning chip-calorimetry measurement on the isothermal crystallization kinetics of PGA in a broad temperature range and compared the results to those of PLLA reported in the literature. The results demonstrate that PGA has the fastest crystallization rate around 130 °C with the minimum crystallization half-time of 4.28 s. The parabolic curve of crystallization half-time versus crystallization temperature of PGA reveals that its crystallization kinetics complies with the classical nucleation theory. The comparison of the crystallization kinetics between PGA and PLLA shows that PGA crystallizes more quickly than PLLA in the whole temperature range, exposing the dominant kinetic factors of their chemical differences in the repeating units. PGA has a much smaller hydrogen side group than the methyl side group of PLLA, thus it has a higher molecular mobility, resulting in a lower glass transition temperature for faster crystallization at low temperatures, as well as a denser molecular packing for a higher melting point and a larger heat of fusion for faster crystallization at high temperatures. This comparison helps us to better understand the basic structure–property relationship in polymer crystallization.

## Data Availability

Data are contained within the article or the Supplementary Materials.

## Supplementary Materials

The following figures are available online at https://www.mdpi.com/2073-4360/13/6/891/s1, Figures S1–S9: (**a**) The temperature dependence of apparent heat capacity of PGA during heating process after isothermal crystallization at (Figure S1) 80 °C for various periods from 1 to 500 s; (Figure S2) 90 °C for various periods from 1 to 300 s; (Figure S3) 100 °C for various periods from 0.01 to 600 s; (Figure S4) 110 °C for various periods from 0.05 to 500 s; (Figure S5) 120 °C for various periods from 0.05 to 600 s; (Figure S6) 140 °C for various periods from 0.01 to 200 s; (Figure S7) 150 °C for various periods from 0.05 to 200 s; (Figure S8) 160 °C for various periods from 1 to 400 s; and (Figure S9) 170 °C for various periods from 1 to 500 s. (**b**) The melting enthalpy evolution curve of PGA after crystallization at the corresponding temperature, obtained from the integration of the melting peaks shown in (**a**).
