# Supplementary material for: Fast-Scanning Chip-Calorimetry Measurement of Crystallization Kinetics of Poly(Glycolic Acid)
Source: Polymers (Basel). 2021 Mar 14;13(6):891. doi: 10.3390/polym13060891 (PMC8001460; doi:10.3390/polym13060891)
Supplement: Supplementary file 1 [file polymers-13-00891-s001.pdf]

Supplementary Materials

Fast-Scanning Chip-Calorimetry Measurement of Crystallization Kinetics of Poly(Glycolic Acid)

Yongxuan Chen, Kefeng Xie, Yucheng He, Wenbing Hu*

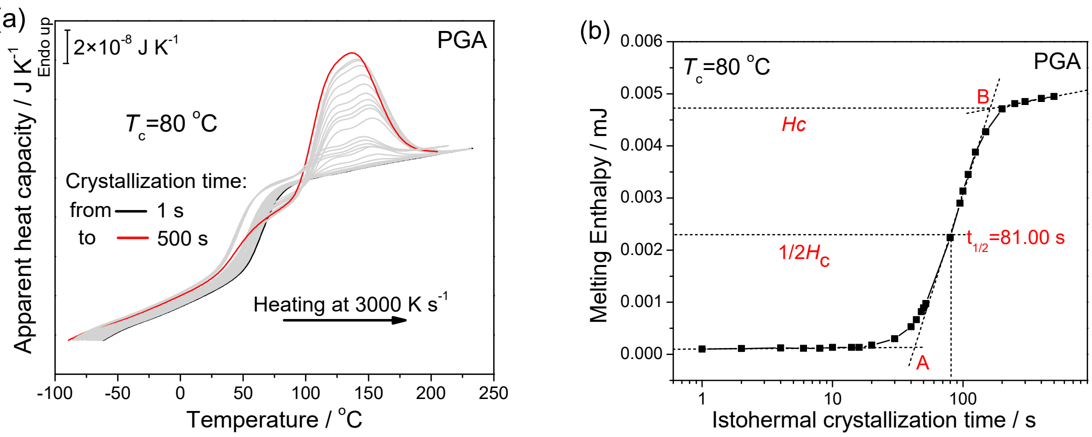

Figure S1. (**a**) the temperature dependence of apparent heat capacity of PGA during heating process after isothermal crystallization at 80 ^{O}C for various periods from 1 to 500 s; (**b**) Crystallization-time evolution of the melting enthalpy of PGA after isothermal crystallization at 80 ^{O}C, obtained from an integration of the melting peaks around 140 ^{O}C shown in **(a)**.

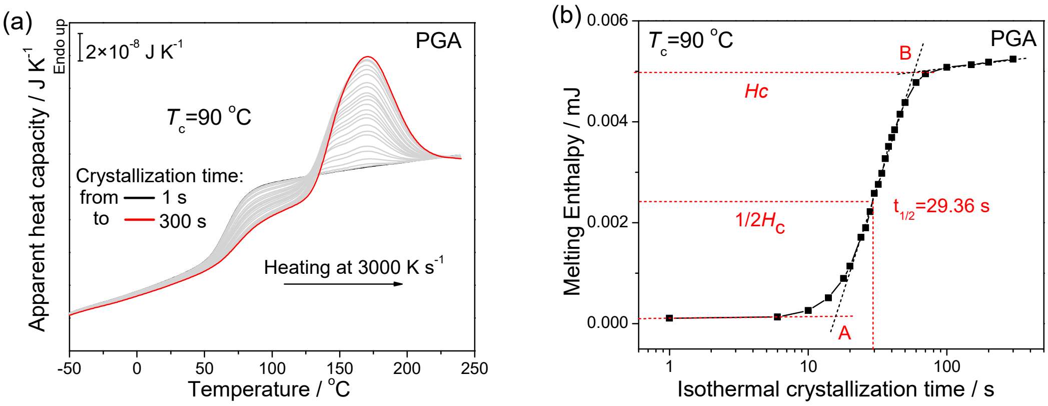

Figure S2. (**a**) the temperature dependence of apparent heat capacity of PGA during heating process after isothermal crystallization at 90 ^{O}C for various periods from 1 to 300 s; (**b**) Melting enthalpy evolution

curve of PGA after crystallization at 90 ^{o}C, obtained from an integration of the melting peaks around 160 ^{O}C shown in **(a)**.

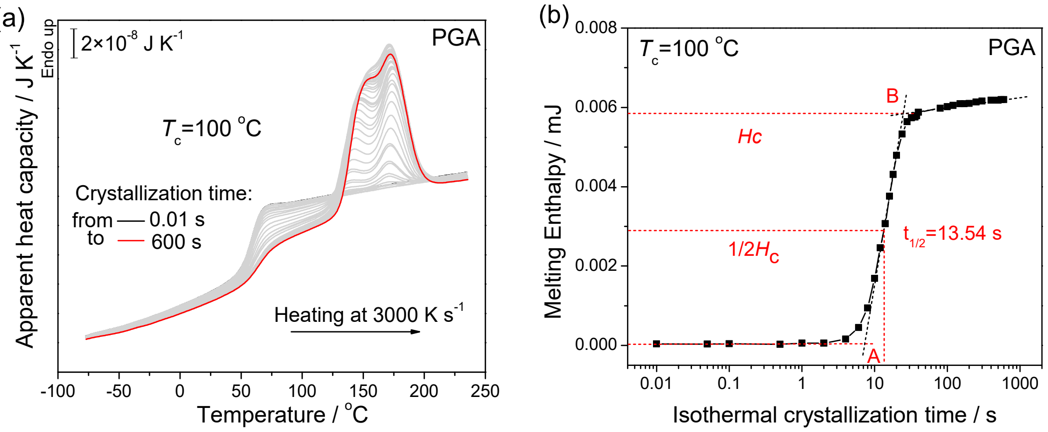

Figure S3. (**a**) the temperature dependence of apparent heat capacity of PGA during heating process after isothermal crystallization at 100 ^{o}C for various periods from 0.01 to 600 s; (**b**) Melting enthalpy evolution curve of PGA after crystallization at 100 ^{o}C, obtained from an integration of the melting peaks around 160 ^{O}C shown in **(a)**.

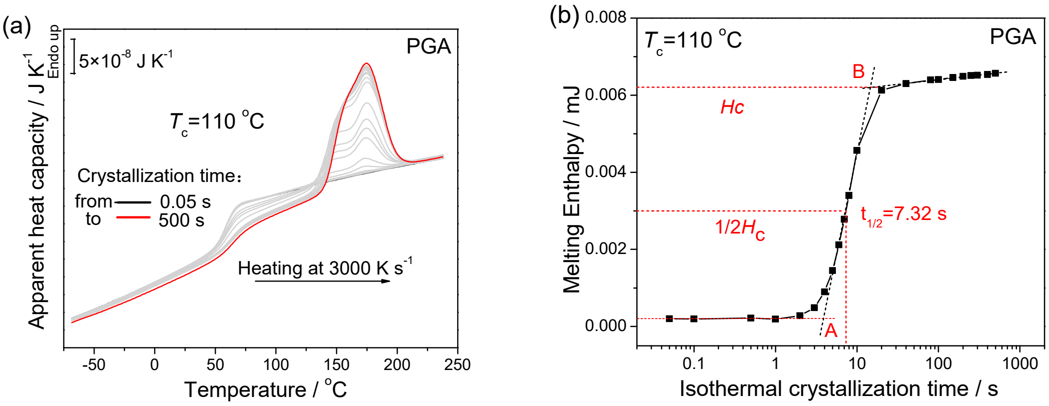

Figure S4. (**a**) the temperature dependence of apparent heat capacity of PGA during heating process after isothermal crystallization at 110 ^{o}C for various periods from 0.05 to 500 s; (**b**) Melting enthalpy evolution curve of PGA after crystallization at 110 ^{o}C, obtained from an integration of the melting peaks around 170 ^{O}C shown in **(a)**.

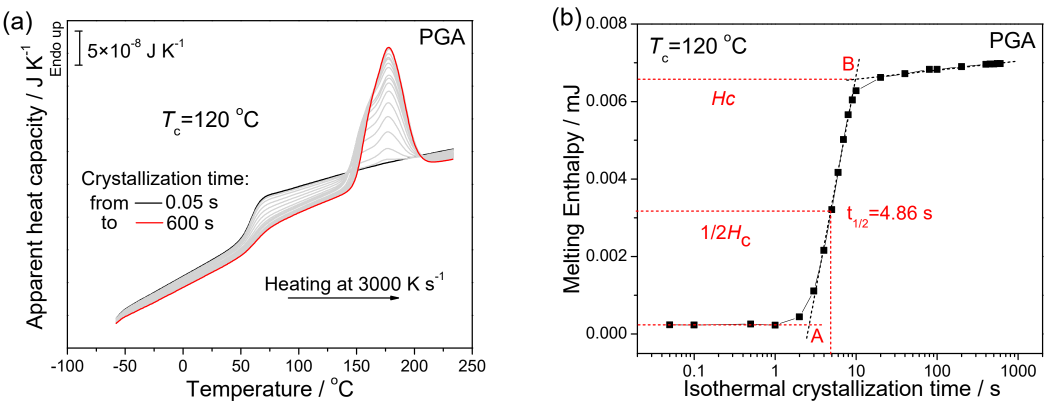

Figure S5. (**a**) the temperature dependence of apparent heat capacity of PGA during heating process after isothermal crystallization at 120 °C for various periods from 0.05 to 600 s; (**b**) Melting enthalpy evolution curve of PGA after crystallization at 120 °C, obtained from an integration of the melting peaks around 170 °C shown in **(a)**.

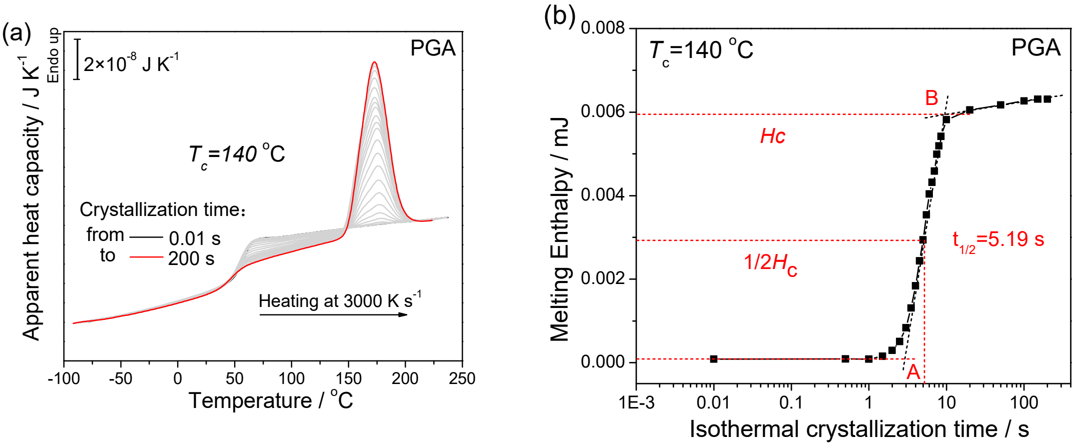

Figure S6. (**a**) the temperature dependence of apparent heat capacity of PGA during heating process after isothermal crystallization at 140 °C for various periods from 0.01 to 200 s; (**b**) Melting enthalpy evolution curve of PGA after crystallization at 140 °C, obtained from an integration of the melting peaks around 180 °C shown in **(a)**.

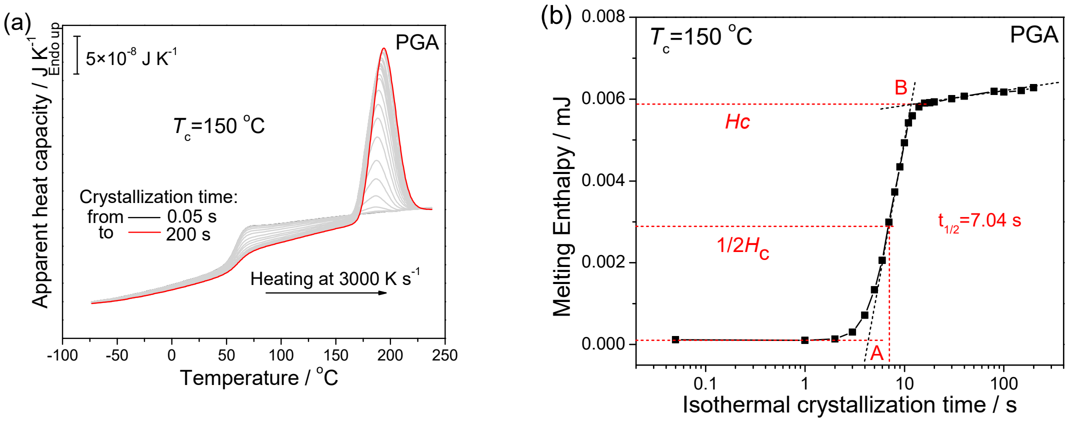

Figure S7. (**a**) the temperature dependence of apparent heat capacity of PGA during heating process after isothermal crystallization at 150 °C for various periods from 0.05 to 200 s; (**b**) Melting enthalpy evolution curve of PGA after crystallization at 150 °C, obtained from an integration of the melting peaks around 190 °C shown in **(a)**.

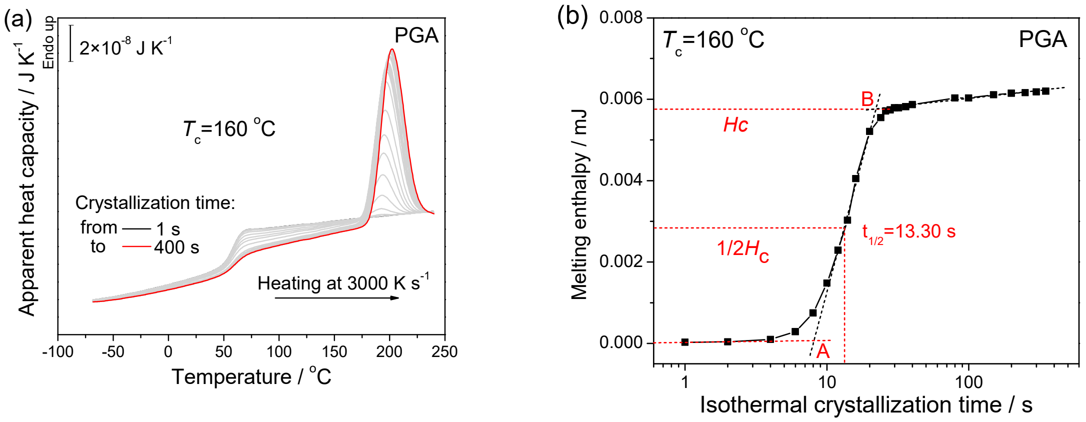

Figure S8. (**a**) the temperature dependence of apparent heat capacity of PGA during heating process after isothermal crystallization at 160 °C for various periods from 1 to 400 s; (**b**) Melting enthalpy evolution curve of PGA after crystallization at 160 °C, obtained from an integration of the melting peaks around 190 °C shown in **(a)**.

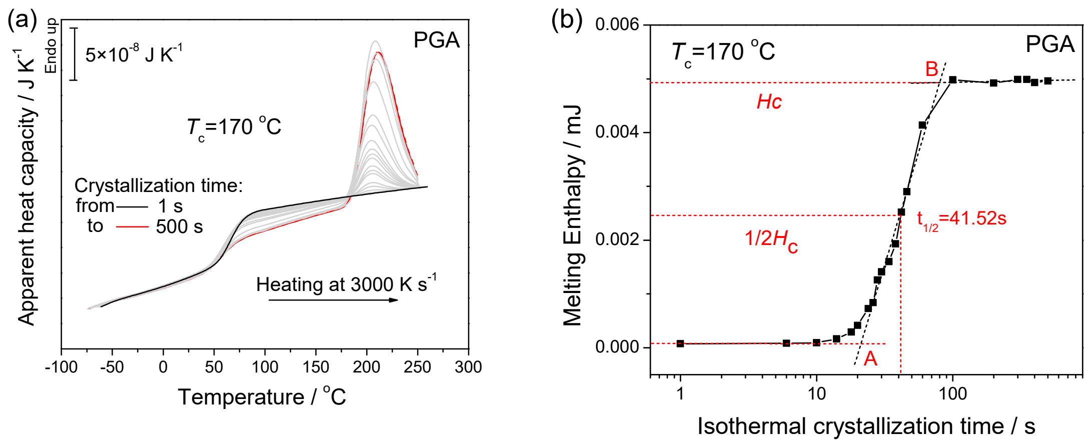

Figure S9. (**a**) the temperature dependence of apparent heat capacity of PGA during heating process after isothermal crystallization at 170 ^{O}C for various periods from 1 to 500 s; (**b**) Melting enthalpy evolution curve of PGA after crystallization at 170 ^{O}C, obtained from an integration of the melting peaks around 200 ^{O}C shown in **(a)**.
